# Molecular substratification of endometrial carcinomas with no special molecular profile (NSMP) by using a limited NGS custom panel may facilitate effective patient selection for the *PIK3CA*-targeted therapy

**DOI:** 10.1007/s00428-024-03905-6

**Published:** 2024-09-05

**Authors:** Ondrej Ondič, Květoslava Michalová, Marián Švajdler, Jiří Presl, Jan Kosťun, Veronika Hájková, Petr Martínek, Michal Michal

**Affiliations:** 1 Department of Pathology, Medical Faculty in Pilsen, Charles University, Prague, Czech Republic; 2 Molecular Genetics Department, Bioptická Laboratoř s.r.o, Pilsen, Czech Republic; 3 Department of Gynecology and Obstetrics, Medical Faculty in Pilsen, Charles University, Prague, Czech Republic

**Keywords:** *PIK3CA*, Kinase, Endometrial carcinoma, Copy number low, Theranostics, Targeted therapy

## Abstract

Endometrial carcinomas (EC) of no special molecular profile (NSMP) represent the largest molecular category of EC, comprising a mixture of tumors with different histology and molecular profiles. These facts likely point to different tumor biology, clinical outcomes, and targeted therapy responses within this molecular category. The *PIK3CA* is currently the only targetable kinase oncoprotein directly implicated in EC carcinogenesis. Investigating a unique single-institution cohort, we attempted to stratify NSMP ECs based on the presence of the PIK3CA pathogenic mutation. Those cases were further analyzed for other well-established-associated oncogenic driver gene mutations. Histological and clinical variables were also correlated in each case. Altogether, 175 ECs were prospectively tested by a limited custom NGS panel containing *ARID1A*,* BCOR*,* BRCA1*,* BRCA2*,* CTNNB1*,* KRAS*,* MLH1*,* MSH2*,* MSH6*,* NRAS*,* PIK3CA*,* PMS2, POLD1*,* POLE*,* PTEN*,and* TP53* genes. We identified 24 *PIK3CA* mutated cases in the group of 80 NSMP ECs, with another co-occurring mutation in at least one oncogenic driver gene (*CTNNB1, PTEN, ARID1A, KRAS, BCOR, PMS2*) in 19 cases. In conclusion, a limited NGS panel can effectively test EC tissue for specific pathogenetically relevant oncogene mutations. The NSMP EC category contains 30% of the *PIK3CA* mutated cases. Of those, 21% contain the *PIK3CA* mutation as a sole EC-associated oncogene mutation, while 79% harbor at least one more mutated gene. These findings may inform future healthcare planning and improve the effectiveness of EC patient selection for the *PIK3CA*-targeted therapy.

## Introduction

The endometrial carcinomas (EC) of no special molecular profile (NSMP) represent the largest molecular category of EC [1]. It comprises a mixture of tumors with different histology and molecular profiles [2, 3]. These facts likely point to different tumor biology, clinical outcomes, and targeted therapy responses within this EC molecular category. The *PIK3CA* is currently the only targetable kinase oncoprotein directly implicated in EC carcinogenesis [1]. Investigating a unique single-institution cohort, we attempted to stratify NSMP ECs based on the presence of the *PIK3CA* pathogenic mutation. Those cases were further analyzed for other well-established EC-associated oncogenic driver gene mutations. Histological and clinical variables were also correlated in each case.

## Material and methods

A retrospective data-drilling study was performed on 175 ECs diagnosed in a single tertiary care hospital from 2020 to June 2023. The cases were prospectively processed in Biopticka laboratory, Pilsen. DNA was extracted from FFPE blocks using a Qiagen DNA mini kit. DNA quantity was measured using Qubit DNA HS (ThermoFisher Scientific, Waltham, MA, USA) after purification using AmpureXp (Beckman Coulter (Brea, CA, USA). The DNA integrity was evaluated using PCR amplification of several control sequences of lengths 100–600 base pairs [4]. Custom panel VariantPlex Biopticka Gyncore Kit (Archer DX, Boulder, CO, USA) was used for mutational analysis of 16 gene targets (ARID1A, BCOR, BRCA1, BRCA2, CTNNB1, KRAS, MLH1, MSH2, MSH6, NRAS, PIK3CA, PMS2, POLD1, POLE, PTEN, TP53) often mutated in endometrial carcinomas. Libraries were constructed using the manufacturer’s protocol, and successfully prepared libraries were sequenced on Nextseq 500/Novaseq 6000 (Illumina, San Diego, CA, USA) with at least 3 million reads per sample, resulting FASTQ files were analyzed using the Archer Analysis software (Archer DX inc.) as described previously [5]. Relevant clinical data in each case was obtained from hospital medical records.

## Results

**Fig. 1:**
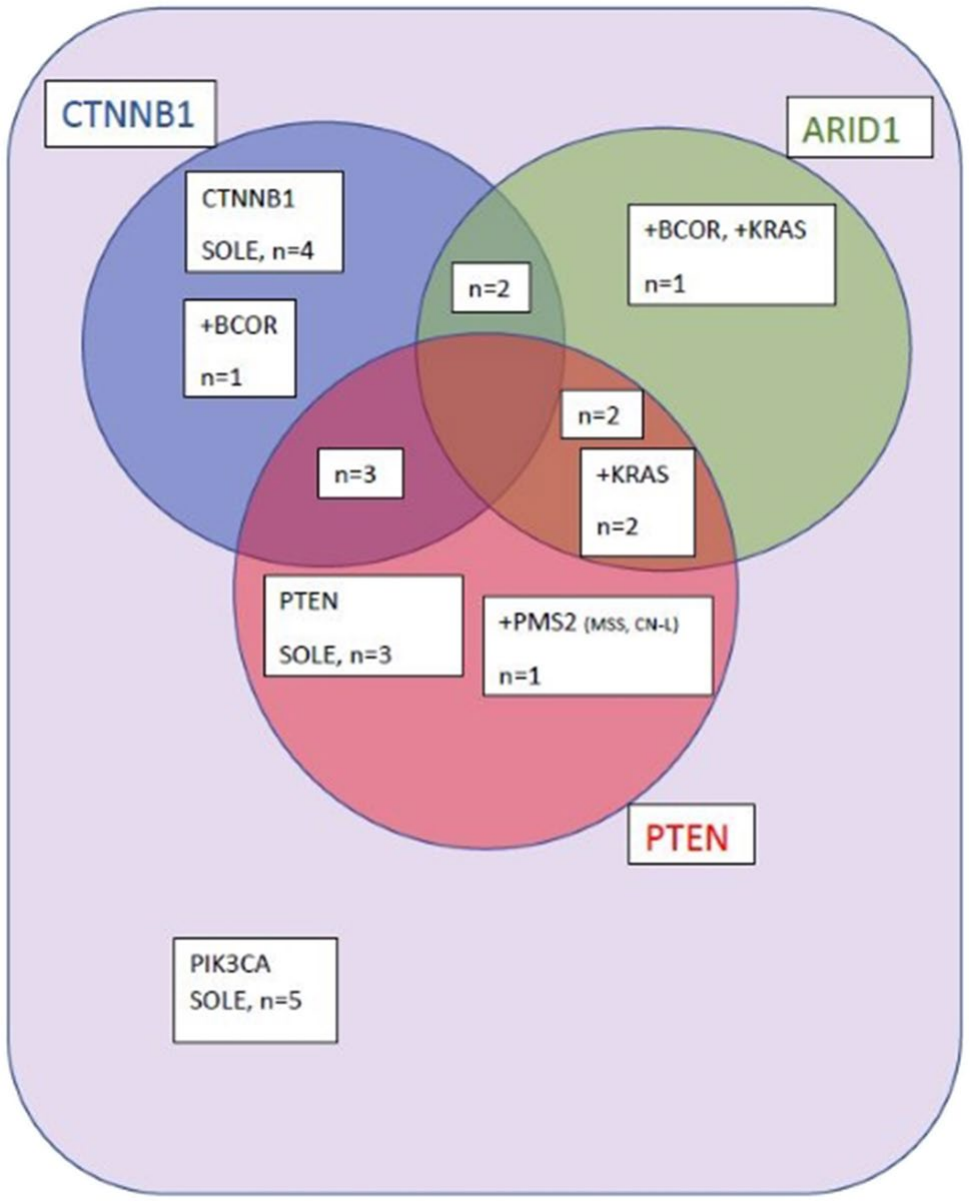
Venn diagram with eight separate areas showing 24 *PIK3CA* mutated endometrial carcinomas of no special molecular pattern (NSMP) divided into seven groups based on co-occurrence of *CTNNB1*,* PTEN*, and *ARID1* gene mutations. Interestingly, five tumors harbor additional mutations in *KRAS*,* BCO*R, and *PMS2* genes, and no tumor simultaneously contained *PIK3CA*, *CTNNB1*, *PTEN*, and *ARID1* mutation

We identified 24 *PIK3CA* mutated cases (Table 1) in women aged 28 to 87 years (mean 62.4, median 63.5 years), representing 30% of the 80 NSMP ECs cohort of women aged 28 to 87 years (mean 62.2, median 63.0 years), with a pathogenic mutation in the *PIK3CA* helical domain in 10 cases and the kinase domain in another 10 cases. Five tumors harbored *PIK3CA* multi-hit mutations affecting the same or different *PIK3CA* gene domains, with two including the linker sequence at codon 118 (Table 1). Of interest, another co-occurring mutation in at least one oncogenic driver gene (*CTNNB1, PTEN, ARID1A, KRAS, BCOR, PMS2*) was present in 19 of 24 cases (79%) (Table 2). The *PIK3CA* as the only mutated driver gene was present in 5 cases (Fig. 1). There was no case harboring simultaneous pathogenic mutations in *PIK3CA, CTNNB1, ARID1A*, and *PTEN* genes (Fig. 1). Overall, 3 *PIK3CA* mutated ECs also contained the *KRAS* mutation paired with *ARID1A* or *PTEN* gene mutation (Fig. 1).

**Table 1:** The PIK3CA gene mutations detected in 24 endometrial cancers with identification of affected protein domains

Case	PIK3CA mut	Domain
1	PIK3CA c.1633G > A, p.(Glu545Lys), AF: 36%, COSM125370	H
2	PIK3CA c.353G > A p.(Gly118Asp) AF: 22% COSM246588; PIK3CA c.3155C > A p.(Thr1052Lys) AF: 27% COSM1220594	K, Li
3	PIK3CA c.3140A > G, p(.His1047Arg), AF: 20%, COSM775	K
4	PIK3CA c.1633G > A, p.(Glu545Lys), AF: 13%, COSM125370	H
5	PIK3CA c.1636C > A p.(Gln546Lys) AF: 14%	H
6	PIK3CA c.1633G > A, p.(Glu545Lys), AF:51%	H
7	PIK3CA c.1624G > A, p.(Glu542Lys), AF: 10%, COSM125369	H
8	PIK3CA c.1133G > A, p.(Cys378Tyr), AF:32%, COSM1041478	C2
9	PIK3CA c.1636C > A, p.(Gln546Lys), AF:41%, COSM766	H
10	PIK3CA c.1624G > A, p.(Glu542Lys), AF: 7%, COSM125369	H
11	PIK3CA c.2176G > A p.(Glu726Lys) AF: 14% COSM87306; PIK3CA c.3139C > T p.(His1047Tyr) AF: 15% COSM1041523	K,K
12	PIK3CA c.353G > A p.(Gly118Asp) AF: 29%	Li
13	PIK3CA c.3140A > G, p.(His1047Arg), AF:24%, COSM775	K
14	PIK3CA c.263G > A, p.(Arg88Gln), AF:29%, COSM746	ABD
15	PIK3CA c.1633G > A, p.(Glu545Lys), COSM125370	H
16	PIK3CA c.3073A > G p.(Thr1025Ala) AF: 10% COSM771	K
17	PIK3CA c.1624G > A, p.(Glu542Lys), AF: 5,15%, COSM125369	H
18	PIK3CA c.1030G > A p.(Val344Met), AF: 9%, COSM253280; PIK3CA c.3061T > A p.(Tyr1021Asn), AF: 43%, COSM6143	C2, K
19	PIK3CA c.1031T > G, p.(Val344Gly), AF:26%, COSM258749	C2
20	PIK3CA c.353G > A, p.(Gly118Asp), AF: 43%, COSM246588; PIK3CA c.1984A > G, p.(Arg662Gly), AF: 7%	Li, H
21	PIK3CA c.3140A > G, p.(His1047Arg), AF:20%, COSM775	K
22	PIK3CA c.3140A > G, p.(His1047Arg), AF: 43,5%, COSM775	K
23	PIK3CA c.3140A > G, p.(His1047Arg), AF: 16%	K
24	PIK3CA c.3140A > T, p.(His1047Leu), AF: 8%, COSM776	K

*ABD* adaptor-binding domain of amino acid (AA) residues 1–108, *Li* linker from AAs 109–190, *C2* domain from AAs 330 to 480, *H* helical domain from AAs 525 to 696, *K* kinase domain from AAs 697 to 1068. Of note, 4 tumors harbor PIK3CA multi-hit mutations affecting the same or different PIK3CA gene domains with two of them including the sequence of domain linker at codon 118

**Table 2:** Thrombotic events, vascular malformations, and co-occurring oncogene mutations in 24 PIK3CA mutated endometrial carcinomas of no special molecular pattern

Case	Mutations	Histology	Stage	Thrombotic event	Vascular malformation
1	*PIK3CA*	E, LG + SQ and HG, gr 2	T1b NX MX	No	No
2	*ARID1A, PIK3CA, CTNNB1*	E, LG, gr 1, SQ	NA	No	Varices
3	*ARID1A, PTEN, PIK3CA*	E, LG, gr 1	NA	No	Hemangiom L3, varices
4	*CTNNB1, PIK3CA*	E, LG, gr 1, SQ	NA	Treated for impaired hemostasis	No
5	*ARID1A, CTNNB1, PIK3CA*	E, LG, gr 1	pT1aN0	*	Lesion of retina
6	*PTEN, ARID1A, PIK3CA*	E, LG, gr 1, SQ	pT1b	No	No
7	*PIK3CA, PTEN*	E, LG, gr 1	T1a	No	No
8	*CTNNB1, PIK3CA, PTEN, PTEN, CTCF*	E, LG, gr 1, SQ	T1a N0	No	No
9	*CTNNB1, PIK3CA*	E, LG, gr 1, SQ	T1a N0	No	No
10	*PIK3CA*	E, LG, gr 1	T1a N0	No	Brain aneurysm surgery
11	*PIK3CA*	E, HG (nuclear), gr 2	T1a N0	Lung embolism	No
12	*ARID1A, PIK3CA, PTEN, KRAS*	E, LG, gr 1, SQ	T1a N0	No	No
13	*PTEN, PIK3CA*	E, LG, gr 1	T1a N0 (0/1 sn)	No	No
14	*ARID1A, KRAS, PIK3CA, PTEN*	E, HG, gr 2	T1a N0 M0	Lung embolism	No
15	*ARID1A, BCOR, KRAS, PIK3CA*	E, LG, gr 1, SQ	T1b	No	Varices
16	*PIK3CA*	E, LG, gr 1	T1b N0	No	Varices
17	*CTNNB1, PIK3CA, PTEN*	E, LG, gr 1	T1b N0	No	No
18	*CTNNB1, PIK3CA*	E, LG, gr 1, SQ	T1b N0 M0	No	No
19	*PIK3CA, PTEN, PMS2*	E, HG, gr2, SQ	T1b N1(mi) (sn)	Leg DVI (2019) + lung embolism	No
20	*PIK3CA*	E, HG, gr 2	T1b2	Thrombosis a. mesenterica sup	No
21	*CTNNB1, PIK3CA, PTEN*	E, LG, gr 1	T2 N0 M0	No	Varices
22	*PIK3CA, PTEN*	E, LG, gr 1	T2 N0 M0	No	No
23	*PIK3CA, CTNNB1*	E, LG, gr 1	T3a	No	No
24	*PIK3CA, CTNNB1, BCOR*	E, LG, gr 1, SQ	T3a N0	No	varices

*DVI* deep vein thrombosis, *NA* not analyzable; * the patient diagnosed with Leiden mutation, *E* endometroid endometrial carcinoma, *SQ* squamous differentiation, *LG* low-grade (WHO 2020), *HG* high grade (WHO 2020), *gr* grade, *varices* clinically managed lower extremity varices minimal grade C2 (CEAP-The 2020 update of the CEAP classification system and reporting standards. Lurie F, 2020, J Vasc Surg Venous Lymphat Disord. 2020 May; 8(3):342–352)

**Fig. 2:**
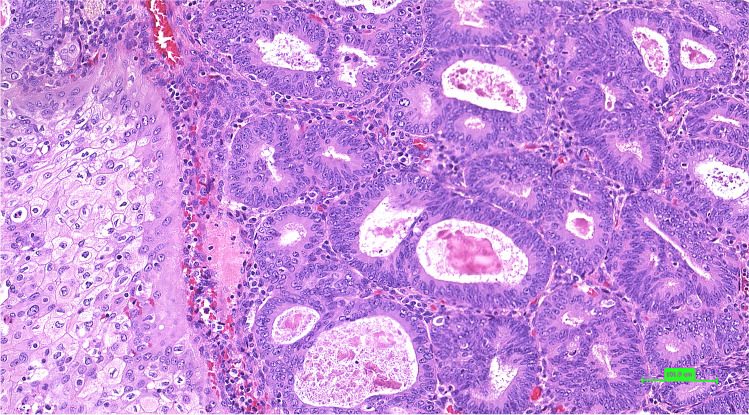
The representative example of endometrial carcinoma of no special molecular pattern (NSMP) harboring the *PIK3CA* mutation presenting the grade 1 (low-grade) histomorphology with squamous morules (case 15)

**Fig. 3:**
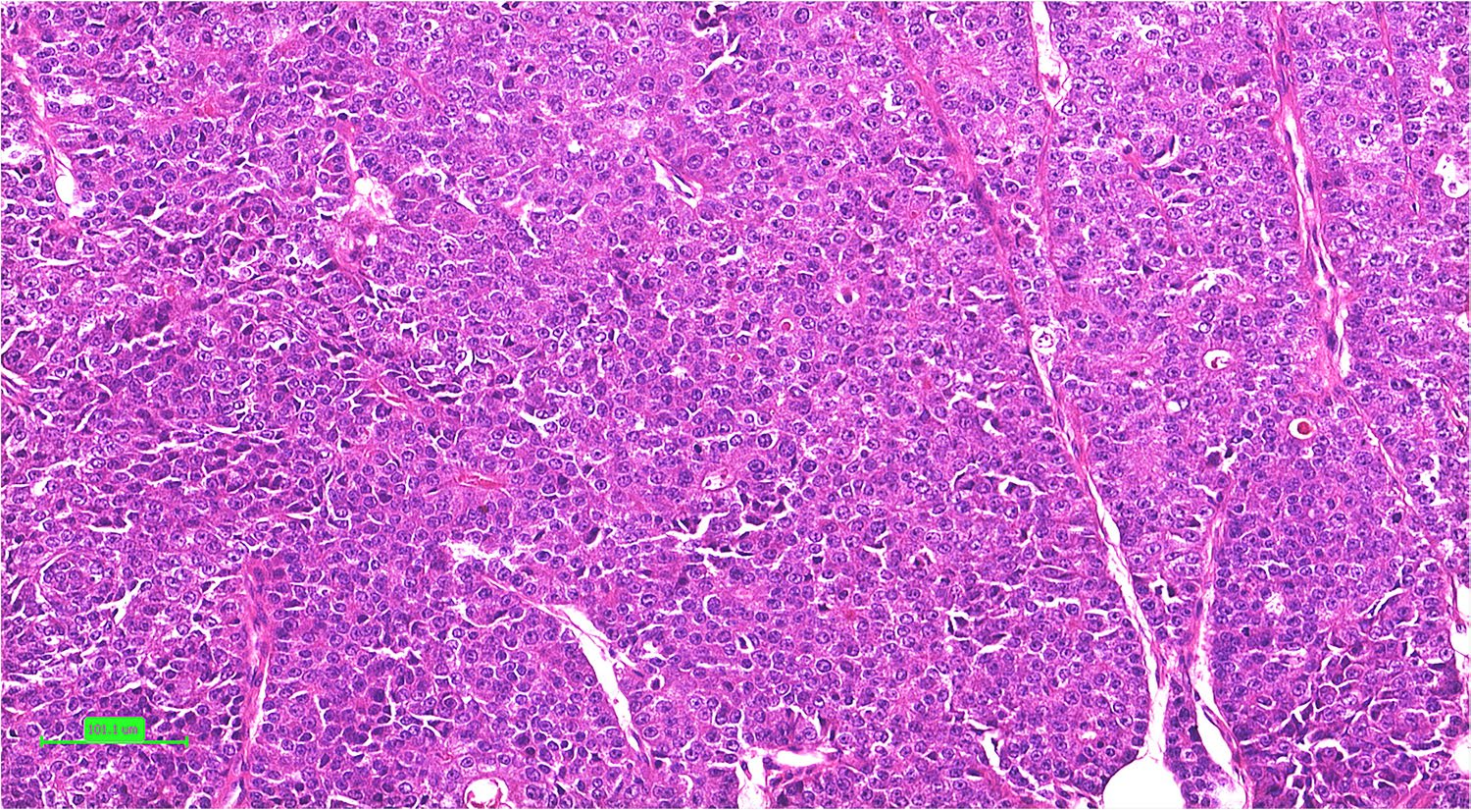
The representative example of endometrial carcinoma of no special molecular pattern (NSMP), harboring the *PIK3CA* mutation presenting high-grade histomorphology (case 19)

Histologically, the study group consisted of 19 low-grade (grade 1) ECs presenting focal squamous differentiation in 9 cases (Fig. 2) and five high-grade ECs (Fig. 3) with focal squamous differentiation in two cases. Interestingly, three of five high-grade ECs contained solo *PIK3CA* mutation. Squamous differentiation corresponded with pathogenic *CTNNB1* mutation in 6 of 19 low-grade cases. Most of the analyzable cases were stage pT1 (16/21), 2 were stage pT2, and 2 were stage pT3 (Table 2).

Eight patients had vascular malformations, including advanced-stage varices of the lower extremities, and five had major thrombotic events (Table 2).

## Discussion

Since 1990, the PIK3CA has been one of the most studied oncogenes, reflecting its frequent mutation in multiple malignancies, including 24 to 46% of ECs [6–13]. Development of PIK3CA inhibitors followed [14, 15], with the treatment of 29 EC patients first reported in 2012 [16]. An additional cohort of 17 patients was reported recently [17, 18]. Two more EC patients are currently treated by PIK3CA inhibitors [19], and another trial is underway [20].

All those trials hint at possible oncologist requests to test ECs for *PIK3CA* mutations in the foreseeable future. Our study focuses on NSMP ECs and shows that a solo *PIK3CA* mutation is present in 21% of cases, making them the most suitable candidates for *PIK3CA* targeted therapy. The *PIK3CA* mutated NSMP ECs (79%) frequently harbor at least one more oncogenic driver gene mutation and can be divided into seven subgroups (Fig. 1), possibly with different targeted therapy response rates. Such cases could be considered candidates for multiple-agent targeted therapy, as suggested recently [2].

Moreover, bioinformatic analysis of individual *PIK3CA* mutation (Table 1), focusing on the domain affected and the presence of multi-hit mutation, may provide another important sub-stratification parameter for analyzing targeted therapy effects in the future.

Of note, personal history in some patients with *PIK3CA* mutated EC encompasses (1) the presence of vascular malformations, including brain vessel aneurysm and frequent clinically advanced varices of lower extremities treated by surgical intervention, and (2) major thrombotic events, including significant pulmonary embolism and thrombosis of the mesenteric artery with intestinal hemorrhagic necrosis and death of the patient (Table 2). These findings may, at least in some cases, be a part of the PIK3CA-related overgrowth spectrum (PROS) syndrome [21, 22], encompassing megalencephaly–capillary malformation (MCAP) syndrome, congenital lipomatous overgrowth, vascular malformations, epidermal nevi, scoliosis/skeletal and spinal (CLOVES) syndrome, fibroadipose overgrowth (FAO), and possibly also the Klippel–Trenaunay syndrome (KTS) and Proteus syndrome (PS). Special clinical recommendations for thrombosis surveillance in PROS have already been proposed [21, 23, 24]. The clinical aspects of the PIK3CA-related overgrowth spectrum syndrome in EC patients should be further investigated. Based on the abovementioned findings and in line with others [2, 3, 18], we argue for more comprehensive molecular testing and bioinformatic analysis of NSMP EC cases beyond the needs defined by the 5th edition of WHO EC molecular classification [25].

## Conclusions

A limited NGS panel can effectively test EC tissue for specific pathogenetically relevant oncogene mutations. The NSMP EC category contains 30% of the *PIK3CA* mutated cases. Of those, 21% contain the *PIK3CA* mutation as a sole EC-associated oncogene mutation, while 79% harbor at least one more mutated gene. These findings may inform future healthcare planning and improve the effectiveness of EC patient selection for the *PIK3CA*-targeted therapy.

## Data Availability

The data that support the findings of this study are available from the corresponding author upon reasonable request.
